# Feeding Preferences for Sugars and Amino Acids in the Red Imported Fire Ant, *Solenopsis invicta* Buren

**DOI:** 10.3390/insects17030258

**Published:** 2026-02-28

**Authors:** Pan Luo, Qing-Xing Shi, Jin-Huan Lou, Ting Chen, Jie Chen, De-Sen Wang, Ming-Yong Ma, Yan Wu, Da-Xing Yang, Guo-Jun Qi

**Affiliations:** 1 Guangdong Provincial Key Laboratory of High Technology for Plant Protection, Plant Protection Research Institute, Guangdong Academy of Agricultural Science, Guangzhou 510640, China; gyu_luopan@163.com (P.L.); shiqingxing@gdppri.com (Q.-X.S.); 19185587149@163.com (J.-H.L.); chent@gdppri.com (T.C.); chenj@gdppri.com (J.C.); 2 Guizhou Provincial Key Laboratory of Agricultural Biosafety, Guiyang University, Guiyang 550005, China; 15150535703@163.com; 3 Department of Entomology, College of Plant Protection, South China Agricultural University, Guangzhou 510642, China; desen@scau.edu.cn; 4 Institute of Plant Protection, Hunan Academy of Agricultural Sciences, Changsha 410125, China; mmyinsect@163.com

**Keywords:** *Solenopsis invicta*, feeding preference, sugars, amino acids, feeding preference assay system

## Abstract

Ant–hemipteran mutualism is mediated by honeydew through long-term coevolution. To clarify the feeding preferences for the main honeydew components (sugars and amino acids) in the red imported fire ant, *Solenopsis invicta* Buren (Hymenoptera: Formicidae), this study employed a self-developed feeding preference assay system to systematically determine the feeding and attraction preferences of workers for ten sugars, eleven amino acids, and their different concentrations and combinations in both laboratory and field experiments. Results showed that: (1) *S. invicta* workers exhibited a significant feeding preference for sucrose and leucine among all tested sugar and amino acid solutions; (2) Feeding preferences for both sucrose and leucine were concentration-dependent. Specifically, preferences for 20% sucrose and 0.5% leucine were significantly higher than for lower concentrations, while not significantly different from higher concentrations; (3) The feeding preference for 10% sucrose or 0.5% leucine alone was higher than that for multi-sugar mixtures or a mixture of all eleven amino acids. However, a mixture of sucrose and leucine solutions significantly enhanced field attraction compared to single solutions of sucrose or leucine. The self-developed feeding preference assay system provides a new tool for measuring feeding preferences in ant colonies under near-natural conditions. Overall, this study systematically clarifies the feeding preference for key nutrients in *S. invicta* workers and establishes a critical theoretical basis for understanding its foraging ecology and developing highly effective liquid baits.

## Simple Summary

Associations between ants (Hymenoptera: Formicidae) and honeydew-producing hemipterans are canonical examples of mutualism in ecosystems. Honeydew, rich in sugars and amino acids, serves as a key factor regulating ant foraging behavior. This study focused on the globally invasive pest, the red imported fire ant, *Solenopsis invicta* Buren. Employing a newly developed feeding preference assay system, laboratory and field experiments were conducted to systematically evaluate the feeding preferences and attraction preference of worker ants for sugars and amino acids, respectively. The results demonstrated that *S. invicta* workers exhibited significantly stronger feeding preferences for sucrose and leucine over other tested compounds. Furthermore, these preferences for both sucrose and leucine intensified with increasing concentration of each compound. Notably, ants preferred single-component sucrose or leucine solutions over multi-component sugar or amino acid mixtures. However, a mixture of sucrose and leucine solutions significantly enhanced the field attraction of *S. invicta* workers. This study clarifies the foraging strategy of *S. invicta* toward key honeydew nutrients and provides a scientific basis for developing efficient and target-specific liquid baits to control fire ants.

## 1. Introduction

Ants and honeydew-producing hemipteran insects have coevolved into a stable and complex mutualistic relationship, which serves as a canonical example of interspecific cooperation in ecosystems [1,2,3,4]. Honeydew, excreted by hemipterans after feeding on plant phloem and xylem sap, is rich in nutrients, particularly sugars and amino acids [5]. For ants, honeydew represents a reliable and abundant carbohydrate resource [6]. In return for this sugar supply, ants protect the hemipterans against natural enemies and parasitoids, and reduce the risk of fungal infection [2,7,8,9]. Ant–hemipteran interactions have potentially broad ecological effects, influencing community structure and ecosystem function, and may even facilitate the co-invasion of invasive alien species [10].

Honeydew-mediated mutualism between ants and honeydew-producing hemipterans is abundant and widespread in arthropod food webs [2,3,7]. Sugars typically constitute over 98% of the dry weight of honeydew [11,12,13], although their specific composition varies among hemipteran species. For example, honeydew from the aphid *Aphis fabae* (Hemiptera: Aphididae) is rich in sucrose, fructose, glucose, and melezitose, with sucrose and fructose predominating [14]. Similarly, honeydew from the cotton mealybug *Phenacoccus solenopsis* (Hemiptera: Pseudococcidae) contains six sugars, primarily sucrose [15]. However, the attractiveness of different sugars to ants varies significantly [14]. Ants generally exhibit a stronger preference for tending hemipteran species whose honeydew is rich in amino acids or specific disaccharides (e.g., sucrose) and trisaccharides (e.g., melezitose) [16]. When multiple honeydew sources are available, ants actively select the most profitable sugar source based on its concentration and composition [17,18], thereby resulting in “selective tending” behavior toward different honeydew-producing hemipteran species.

In nature, ants not only forage for solid foods such as insects to supplement protein [19] but also consume liquid food sources, including honeydew and extrafloral nectar, to obtain carbohydrates [20]. A carbohydrate-rich diet can prolong an individual’s lifespan but may reduce an individual’s reproductive capacity. In contrast, a protein-rich diet is more favorable for colony reproduction [21,22]. These nutritional preferences exhibit distinct caste specificity within the ant colony: workers primarily rely on carbohydrates for energy, whereas queens and larvae typically require more protein to support egg-laying and rapid growth [23]. Furthermore, foraging preferences diverge by niche: arboreal ants typically prioritize protein acquisition, whereas terrestrial ants commonly favor carbohydrates [24]. Therefore, complex solutions containing amino acids, proteins, and other nutrients can more holistically address the diverse nutritional needs of ant colonies across different ecological niches and colony development than simple sugar solutions.

Current laboratory assays for evaluating sugar feeding preferences in the red imported fire ant commonly involve separating individuals from their soil nests using the “water-flotation method,” before conducting bridge or filter-paper assays [25,26]. However, such approaches may disrupt the natural behavior of ants [27]. In particular, for soil-dwelling species, removal from their native soil environment may alter foraging decisions, thereby compromising the ecological validity of behavioral observations [28]. Furthermore, under field conditions, *S. invicta* workers frequently transport small stones, sand particles, and dead leaves into food sources [27], severely interfering with accurate measurements and rendering direct measurement of food consumption impractical. To minimize anthropogenic disturbance and more closely approximate natural conditions, we designed and employed a self-developed feeding preference assay system that retained the ants’ original soil habitat. In this study, we focused on the globally invasive pest, the red imported fire ant, *Solenopsis invicta* Buren. Laboratory and field experiments were conducted to systematically evaluate the feeding and attraction preferences of worker ants for sugars and amino acids by using this self-developed feeding preference assay system. Our study addressed three main objectives: (1) to clarify the preference hierarchy of workers for different sugars and amino acids; (2) to elucidate the effects of sugar and amino acid concentrations on feeding preference; and (3) to investigate potential synergistic effects of sugar-amino acid mixtures. This research aims to characterize the feeding-behavioral responses of *S. invicta* to key nutrients, thereby providing a theoretical basis for developing effective liquid baits and enhancing control strategies.

## 2. Materials and Methods

### 2.1. Insect Colonies

Colonies of *S. invicta* were collected from Aotou Town (23°37′12″ N, 113°25′22″ E, 37 m), Conghua District, Guangzhou City, Guangdong Province, China. Field collection followed the method described by Chen [29], with a minimum distance of 50 m between selected nests. After collection, colonies were allowed to acclimate and establish new nests in plastic containers (40 cm length, 30 cm width, and 23 cm height) for 2–3 days. Subsequently, each stabilized colony was transferred to a large plastic basin (50 cm length, 40 cm width, and 15 cm height) and gently subdivided into five subcolonies using a small shovel. Each sub-colony contained at least two queens, ten alates, and a substantial number of workers, eggs, larvae, and pupae, along with the original nest soil. These subcolonies were placed into separate plastic buckets (24 cm diameter, 20 cm height). The soil surface was leveled by gently shaking the bucket, and the feeding preference assay system (described in Section 2.3) was immediately inserted vertically into the center, ensuring it remained level. A 10 mL centrifuge tube filled with distilled water and plugged with cotton was provided on the platform of the assay system for hydration. Prior to experimentation, colonies were maintained for seven days on a diet of 10% (*v*/*v*) honey water (COFCO Shancui Natural Foods Co., Ltd., Beijing, China) and larvae of the yellow mealworm, *Tenebrio molitor* L. Ant colonies were maintained under controlled conditions of 26 ± 2 °C, approximately 75% relative humidity (RH), and a 12 h light: 12 h dark photoperiod.

### 2.2. Chemicals and Reagents

Ten common sugars were used in this study including D-(+)-glucose, D-(+)-maltose monohydrate, D-(+)-xylose, D-(-)-fructose (Shanghai Biorigin Biotechnology Co., Ltd., Shanghai, China), sucrose (Guangzhou Chemical Reagent Factory Co., Ltd., Guangzhou, China), lactose monohydrate (Tianjin Comio Chemical Reagent Co., Ltd., Tianjin, China), D-(-)-ribose, D-(+)-galactose, D-(+)-trehalose dihydrate (Shandong Keyuan Biochemical Co., Ltd., Heze, China), and D-(+)-melezitose hydrate (Shanghai Aladdin Biochemical Technology Co., Ltd. Shanghai, China).

Eleven L-amino acids were selected based on a previous study [30]: L-leucine, L-threonine, L-arginine, L-isoleucine, L-histidine, L-methionine, L-phenylalanine, L-glutamic acid, L-tryptophan, L-alanine, and L-valine (Shanghai Macklin Biochemical Technology Co., Ltd., Shanghai, China). All reagent solutions were prepared each day freshly using deionized water.

### 2.3. Feeding Preference Assay System for Ants

**Figure 1:**
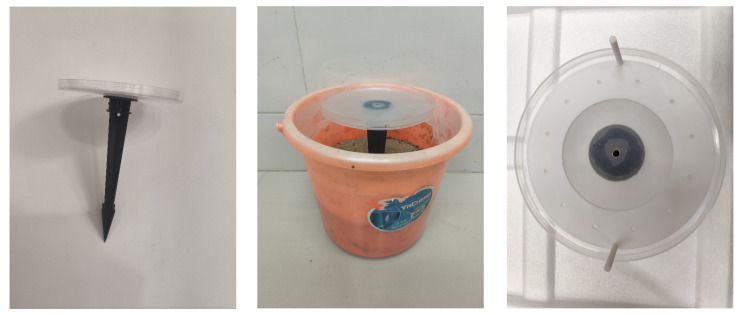

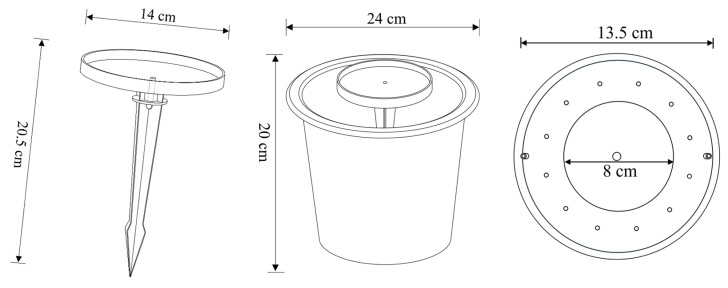
Photograph (**top**) and schematic diagram (**bottom**) of the feeding preference assay system for ants.

A feeding preference assay system was developed to minimize disturbance to ant colonies. The system consisted of three main components: a plastic bucket (24 cm diameter, 20 cm height) housing a sub-colony, an insertion rod (20.5 cm height), and a circular foraging platform (modified from a 14 cm diameter Petri dish). To facilitate ant climbing, the Petri dish bottom and the insertion rod surface were roughened with 400-grit sandpaper. A 4.0 mm hole in the top of the insertion rod was aligned with a 5.0 mm hole in the center of the dish and permanently fixed with hot glue, forming an elevated and independent foraging arena. Foraging workers could exit the nest in the bucket, climb the insertion rod, and enter the platform through the connected holes to make feeding choices (Figure 1).

Furthermore, to ensure synchronous access to treatment solutions and consistent travel distance for foraging workers, a testing frame (outer diameter: 13.5 cm, inner diameter: 8 cm) equipped with two cylindrical handles (0.3 cm diameter, 5 cm height) was placed concentrically on the platform. The 12 small holes on the testing frame are equidistant positioning markers for alignment, and the number of feeding dishes varies by experiment (Figure 1). Feeding dishes, made from 10 mL centrifuge tube lids (1.3 cm diameter, 0.5 cm height) were positioned on the testing frame. The frame was then positioned concentrically on the circular foraging platform, ensuring an equal distance from the central entry point of the platform to each feeding dish.

### 2.4. Experimental Procedures

In laboratory experiments, all subcolonies housed in buckets were deprived of food for 48 h while maintaining access to water prior to each preference test. To eliminate potential interference from residual trail pheromone, the foraging platform surface was thoroughly wiped with a damp paper towel 10 min before testing. All experiments were conducted under controlled conditions at 26 ± 2 °C and approximately 75% relative humidity. The detailed procedure for the feeding preference assay was as follows: Feeding dishes were randomly placed on the testing frame. The frame was first weighed using an electronic balance (EXD-322B, Putian Hengke Instrument Co., Ltd., Fujian, China) and the initial mass was recorded. A micropipette (BioPette™ Plus, Labnet International Inc., Woodbridge, NJ, USA) was used to dispense 0.5 mL of each test solution into its assigned dish, after which the weight of each dish containing the solution was recorded. The frame was then centered on the foraging platform. An overhead camera (Redmi Note 13 Pro, Xiaomi Technology Co., Ltd., Beijing, China) recorded the platform continuously for 60 min. At 5 min intervals, the number of workers actively feeding at each solution was counted. An ant was recorded as feeding if its head was lowered and its mouthparts were in contact with the liquid. After 60 min, the frame with the dishes was re-weighed, and the consumption of each solution was calculated. Five independent colony replicates were performed. In each replicate, all five subcolonies were tested simultaneously, and the positions of the treatments on the frame were randomized to control for potential location bias.

**Figure 2:**
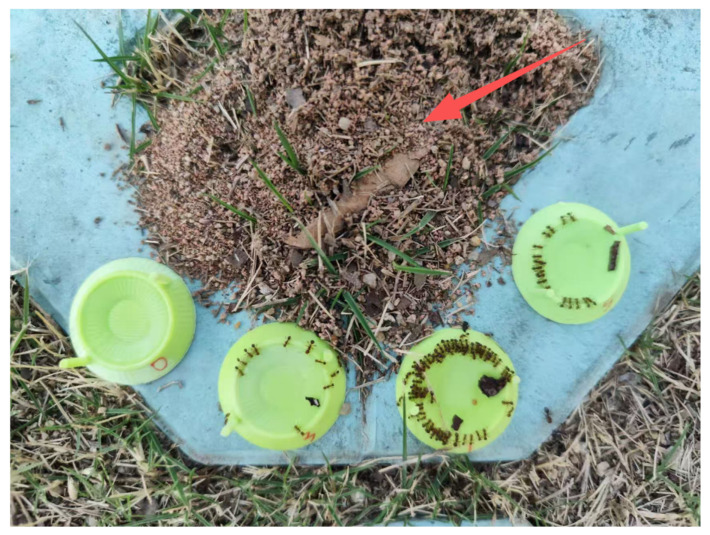
Field-based assessment of attraction preferences. The red arrow indicates the location of an *S. invicta* nest.

In field experiments, the study site was located near the Dongchong subway station (22°53′09″ N, 113°28′45″ E, 21 m), Nansha District, Guangzhou City, an area free of insecticides for at least one month prior to the experiment. The solution container was a 3D-printed concentric circular depression dish (2 cm outer diameter, 1 cm inner diameter, 0.5 cm height) equipped with two cylindrical handles (0.2 cm diameter, 1 cm height). A volume of 1 mL of each test solution was dispensed into a dish using a micropipette. Dishes were randomly arranged around five independent ant nests (Figure 2), with nests spaced more than 10 m apart. Observations were made on sunny days between 13:00 and 17:00. To avoid measurement errors in solution volume caused by capillary action or transported debris [27], field attraction preference was quantified solely as the number of worker ants recruited to each solution, rather than by consumption. Photographs taken 2 h after solution placement were used to count the number of workers feeding at each dish.

#### 2.4.1. Evaluation of Feeding Preference for Sugars and Amino Acids

Based on the solubility of sugars and amino acids in water, test solutions were formulated at concentrations of 10% (*w*/*v*) for each sugar and 0.5% (*w*/*v*) for each amino acid. Deionized water served as a control. For each assay, 0.5 mL aliquots of each test solution were dispensed in laboratory experiments, whereas 1 mL aliquots were used in field experiments. The feeding preference and field attraction preference of *S. invicta* workers were then evaluated following the protocol described above.

#### 2.4.2. Evaluation of Feeding Preference for Different Concentration Gradients

Based on the preliminary results, sucrose and leucine were selected as the target compounds for concentration–response evaluation. A series of sucrose solutions was formulated at concentrations of 5%, 10%, 20%, 40%, and 70% (*w*/*v*). Similarly, L-leucine solutions were formulated at concentrations of 0.125%, 0.25%, 0.5%, 1%, and 2% (*w*/*v*). Deionized water served as a control. For each assay, 0.5 mL aliquots of each test solution were dispensed in laboratory experiments, whereas 1 mL aliquots were used in field experiments. The feeding preference and field attraction preference of *S. invicta* workers were then evaluated following the protocol described above.

#### 2.4.3. Evaluation of Feeding Preference for Combination Solutions

Based on the preliminary feeding preference results, the test solution sets were as follows: (1) Sugars: 10% (*w*/*v*) sucrose, a 10% “Top 3” sugar mixture (sucrose: melezitose: glucose = 1:1:1 *w*/*w*), and a 10% “Top 5” sugar mixture (sucrose: melezitose: glucose: maltose: fructose = 1:1:1:1:1 *w*/*w*); (2) Amino acids: 0.5% (*w*/*v*) leucine and a 0.5% (*w*/*v*) equimass mixture of all eleven amino acids; (3) Sugar–amino acid mixture: 10% (*w*/*v*) sucrose, 0.5% (*w*/*v*) leucine, and a combined solution of 10% (*w*/*v*) sucrose + 0.5% (*w*/*v*) leucine. Deionized water served as a control. For each assay, 0.5 mL aliquots of each test solution were dispensed in laboratory experiments, whereas 1 mL aliquots were used in field experiments. The feeding preference and field attraction preference of *S. invicta* workers were then evaluated following the protocol described above.

### 2.5. Data Analysis

To account for variations in the number of foraging workers across subcolonies in the laboratory experiments and among nests in the field experiments, individual ant counts were standardized to relative feeding intensity for subsequent analysis [31]. In the laboratory experiments, the relative feeding intensity at a given time point was calculated as the proportion of ant workers feeding on a target solution relative to the total number of ant workers feeding on all solutions at that time point. In the field experiments, the relative attraction intensity was calculated as the proportion of ant workers at a target solution relative to the total number of ant workers observed at all solutions. Relative feeding intensity and relative attraction intensity data were arcsine square-root transformed before analysis. One-way analysis of variance (ANOVA) was performed using IBM SPSS Statistics 27.0 (IBM Corp., Armonk, NY, USA), with Tukey’s HSD test applied for multiple comparisons. For all tests, the significance level was set at *α* = 0.05.

## 3. Results

### 3.1. Feeding Preference of S. invicta Workers for Sugars and Amino Acids

**Figure 3:**
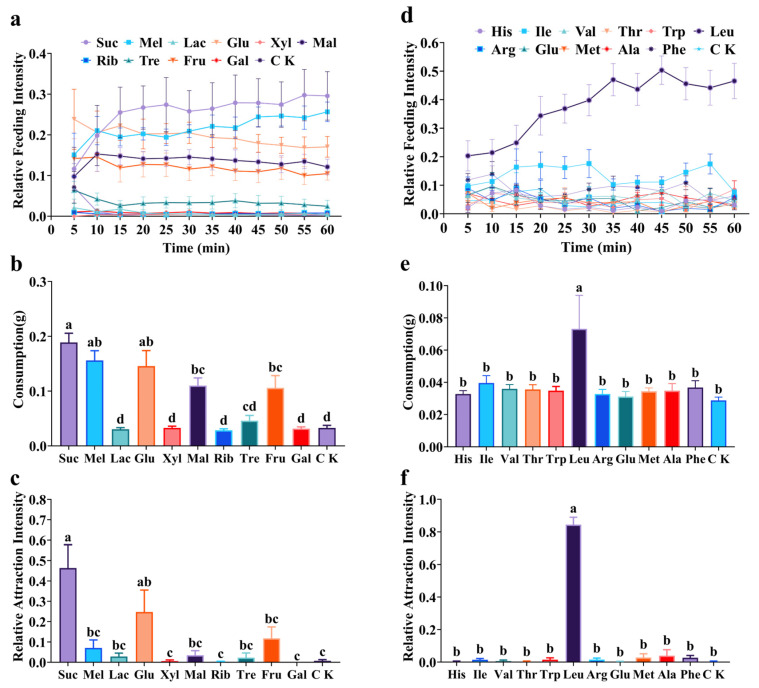
Feeding preference of *S. invicta* workers for different sugars (**left**) and different amino acids (**right**). (**a**,**d**) Relative feeding intensity (laboratory); (**b**,**e**) Consumption (laboratory); (**c**,**f**) Relative attraction intensity (field). Notes: Data are presented as mean ± SE (*n* = 5). Different lowercase letters above bars indicate significant differences (Tukey’s HSD test, *p* < 0.05). Abbreviations: Suc: sucrose; Mel: melezitose; Lac: lactose; Glu: glucose; Xyl: xylose; Mal: maltose; Rib: ribose; Tre: trehalose; Fru: fructose; Gal: galactose; His: histidine; Ile: isoleucine; Val: valine; Thr: threonine; Trp: tryptophan; Leu: leucine; Arg: arginine; Glu: glutamic acid; Met: methionine; Ala: alanine; Phe: phenylalanine; CK: deionized water.

*Solenopsis invicta* workers exhibited significant differences in their feeding preferences among ten different sugars (Figure 3a–c). In laboratory experiments, the relative feeding intensity of *S. invicta* fluctuated considerably during the first 15 min. Subsequently, the feeding intensity on sucrose, melezitose, glucose, maltose, and fructose remained at a relatively high level, with sucrose consistently eliciting the strongest response (Figure 3a). At the 60 min time point, feeding intensity for sucrose and melezitose was significantly higher than for lactose, xylose, maltose, ribose, trehalose, fructose, and galactose, but not significantly different from glucose (*F* = 38.24, df = 10, *p* < 0.0001). After 60 min, total consumption also differed significantly among sugars (*F* = 17.89; df = 10; *p* < 0.0001). Sucrose consumption was the highest and not significantly different from that of melezitose and glucose. Consumption of sucrose, melezitose, and glucose was significantly greater than that of lactose, xylose, ribose, trehalose, and galactose (Figure 3b). Field experiments corroborated the laboratory findings. Sucrose also exhibited the strongest field attraction for *S. invicta*, which was not significantly different from glucose but was significantly higher than for the other eight sugars (*F* = 7.98; df = 10; *p* < 0.0001) (Figure 3c).

There were also significant differences in the feeding preferences of *S. invicta* towards eleven different amino acids (Figure 3d–f). In the laboratory experiments, the relative feeding intensity for leucine consistently remained the highest and increased gradually over time (Figure 3d). At the 60 min time point, feeding intensity for leucine was significantly higher than that for the other ten amino acids (*F* = 14.94, df = 11, *p* < 0.0001). Similarly, final consumption after 60 min differed significantly among amino acids (*F* = 2.95, df = 11, *p* < 0.0001), with leucine consumption being the highest and significantly exceeding that of all the other ten amino acids (Figure 3e). Field experiments strongly supported this distinct preference. Leucine elicited significantly greater attraction than the other ten amino acids (*F* = 38.02, df = 11, *p* < 0.0001) (Figure 3f).

### 3.2. Feeding Preference of S. invicta Workers for Different Concentration Gradients

**Figure 4:**
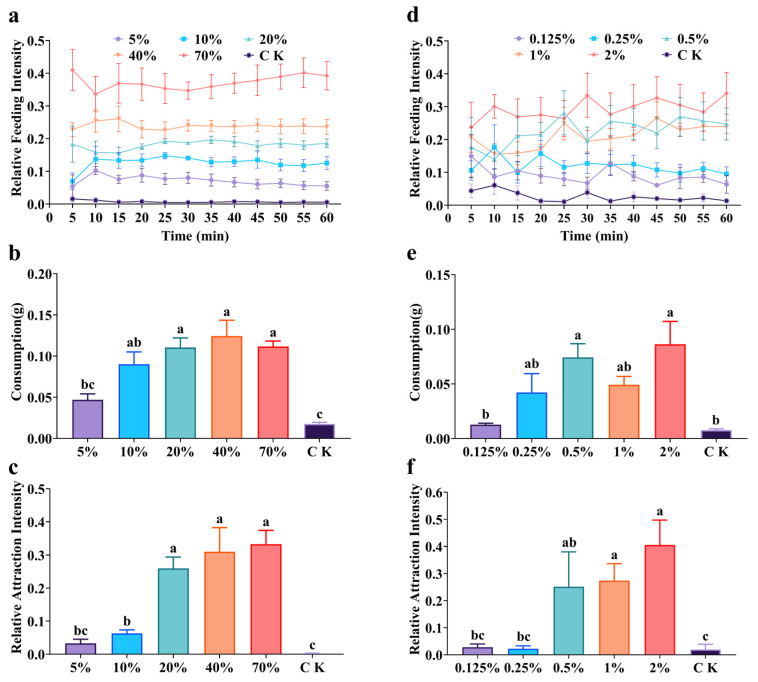
Feeding preference of *S. invicta* workers for sucrose (**left**) and leucine (**right**) at different concentrations. (**a**,**d**) Relative feeding intensity (laboratory); (**b**,**e**) Consumption (laboratory); (**c**,**f**) Relative attraction intensity (field). Notes: Data are presented as mean ± SE (*n* = 5). Different lowercase letters above bars indicate significant differences (Tukey’s HSD test, *p* < 0.05). Abbreviations: 5%: 5% sucrose; 10%: 10% sucrose; 20%: 20% sucrose; 40%: 40% sucrose; 70%: 70% sucrose; 0.125%: 0.125% leucine; 0.25%: 0.25% leucine; 0.5%: 0.5% leucine; 1%: 1% leucine; 2%: 2% leucine; CK: deionized water.

The feeding preference of *S. invicta* workers for sucrose increased with concentration (Figure 4a–c). In the laboratory experiments, higher sucrose concentrations elicited greater relative feeding intensity (Figure 4a). At the 60 min time point, the feeding intensity for 70% sucrose was significantly higher than for concentrations ranging from 5% to 40% (*F* = 52.08, df = 5, *p* < 0.0001). Consumption after 60 min also varied significantly with sucrose concentrations (*F* = 12.95, df = 5, *p* < 0.0001). Consumption was highest for the 40% sucrose solution, which was significantly greater than for the 5% solution but not significantly different from the 10%, 20%, and 70% solutions (Figure 4b). Field experiments strongly supported these laboratory findings. The relative attraction for 20% sucrose was not significantly different from the 40% and 70% solutions but was significantly higher than the 5% and 10% solutions (*F* = 27.90, df = 5, *p* < 0.0001) (Figure 4c).

A clear concentration-dependent response was also observed in their feeding preference for leucine (Figure 4d–f). In the laboratory experiments, higher leucine concentrations generally elicited greater relative feeding intensity (Figure 4d). At the 60 min time point, the feeding intensity for 2% leucine showed no significant difference from the 1% and 0.5% solutions but was significantly higher than the 0.25% and 0.125% solutions (*F* = 16.43, df = 5, *p* < 0.0001). After 60 min, consumption likewise differed significantly across the leucine concentrations (*F* = 6.35, df = 5, *p* < 0.001). Consumption was highest for the 2% leucine solution, being significantly greater than that for the 0.125% solution but not significantly different from the 0.25%, 0.5%, and 1% solutions (Figure 4e). Field experiments confirmed this trend. The 2% leucine solution elicited the highest relative attraction, which did not differ significantly from the 0.5% and 1% solutions but was significantly higher than the 0.125% and 0.25% solutions (*F* = 8.98, df = 5, *p* < 0.05) (Figure 4f).

### 3.3. Feeding Preference of S. invicta Workers for Combination Solutions

**Figure 5:**
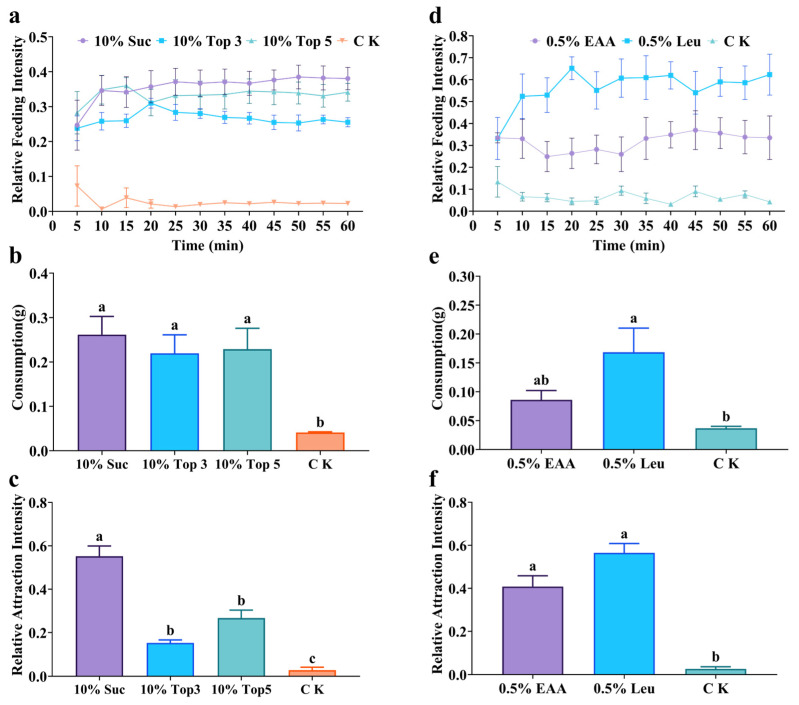
Feeding preference of *S. invicta* workers for sugar mixtures (**left**) and amino acid mixtures (**right**). (**a**,**d**) Relative feeding intensity (laboratory); (**b**,**e**) Consumption (laboratory); (**c**,**f**) Relative attraction intensity (field). Notes: Data are presented as mean ± SE (*n* = 5). Different lowercase letters above bars indicate significant differences (Tukey’s HSD test, *p* < 0.05). Abbreviations: 10% Suc: 10% sucrose; 10% Top 3: 10% mixture of sucrose, melezitose, and glucose; 10% Top 5: 10% mixture of sucrose, melezitose, glucose, maltose, and fructose; 0.5% Leu: 0.5% leucine; 0.5% EAA: 0.5% mixture of histidine, isoleucine, valine, threonine, tryptophan, leucine, arginine, glutamic acid, methionine, alanine, and phenylalanine; CK: deionized water.

*Solenopsis invicta* workers displayed a stronger feeding preference for a single 10% sucrose solution than for multi-sugar mixtures (Figure 5a–c). In the laboratory experiments, the relative feeding intensity for sucrose alone remained high, while mixtures containing melezitose, glucose, or other sugars did not enhance attraction (Figure 5a). At the 60 min time point, feeding intensity for single sucrose was significantly higher than for the Top 3 mixture but not significantly different from the Top 5 mixture (*F* = 93.72, df = 3, *p* < 0.0001). After 60 min, consumption of single sucrose did not differ significantly from either the Top 3 or Top 5 mixture but was significantly higher than the control (*F* = 7.04, df = 3, *p* < 0.05) (Figure 5b). Importantly, field experiments revealed a clear attraction for single sucrose, which attracted significantly more ant workers than both mixtures (*F* = 51.43, df = 3, *p* < 0.0001) (Figure 5c).

Similarly, a single 0.5% leucine solution was preferred over a mixture of all eleven amino acids (Figure 5d–f). In the laboratory experiments, leucine alone consistently sustained the highest relative feeding intensity (Figure 5d). At the 60 min time point, the feeding intensity for the single leucine solution showed no significant difference from the amino acid mixture (*F* = 17.62, df = 2, *p* < 0.001). After 60 min, consumption of single leucine did not differ significantly from the amino acid mixture but was significantly higher than the control (*F* = 6.57, df = 2, *p* < 0.05) (Figure 5e). Field experiments strongly supported these laboratory findings. Both single leucine and the mixture of all amino acids attracted significantly more ant workers than the control, with no significant difference between them (*F* = 64.77, df = 2, *p* < 0.0001) (Figure 5f).

**Figure 6:**
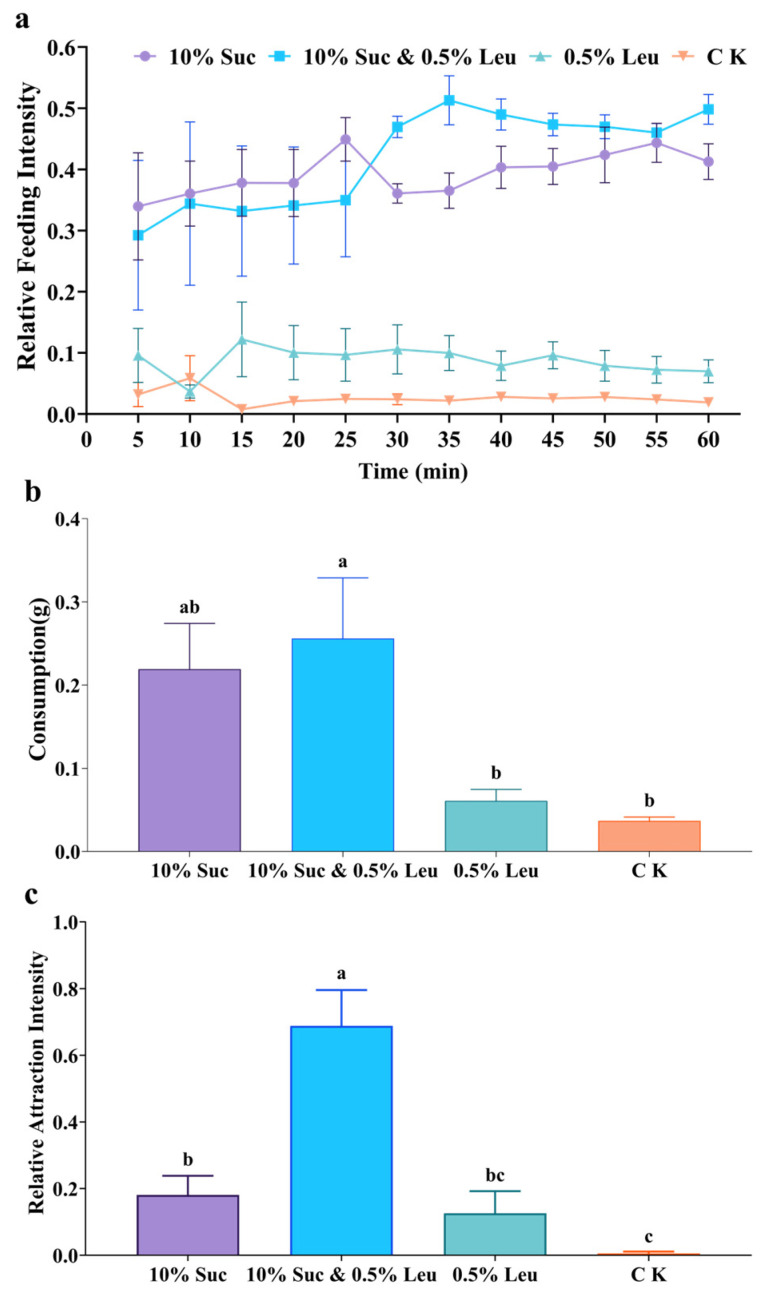
Feeding preference of *S. invicta* workers for sugar-amino acid mixtures. (**a**) Relative feeding intensity (laboratory), (**b**) Consumption (laboratory); (**c**) Relative attraction intensity (field). Notes: Data are presented as mean ± SE (*n* = 5). Different lowercase letters above bars indicate significant differences (Tukey’s HSD test, *p* < 0.05). Abbreviations: 10% Suc: 10% sucrose; 0.5% Leu: 0.5% leucine; 10% Suc and 0.5% Leu: 10% sucrose and 0.5% leucine; CK: deionized water.

Notably, a mixture of 10% sucrose and 0.5% leucine exhibited a stronger feeding preference than either 10% sucrose or 0.5% leucine alone, particularly under field conditions (Figure 6). In the laboratory experiments, the feeding intensity for the sucrose-leucine mixture was slightly lower than for single sucrose at the beginning but surpassed it distinctly after 30 min (Figure 6a). At the 60 min time point, the feeding intensity for the mixture was significantly higher than for leucine alone but showed no significant difference from that for sucrose alone (*F* = 107.8, df = 3, *p* < 0.0001). After 60 min, consumption differed significantly among the solutions (*F* = 5.71, df = 3, *p* < 0.05). The mixture’s consumption was significantly greater than for leucine alone but not significantly different from sucrose alone (Figure 6b). The synergistic effect was most pronounced in the field experiments, where the sucrose-leucine mixture elicited significantly greater attraction than either single-component solution (*F* = 20.93, df = 3, *p* < 0.0001) (Figure 6c).

## 4. Discussion

Ants frequently consume liquid foods such as honeydew and extrafloral nectar to meet their carbohydrate requirements [20], a foraging strategy shaped by their physiology, nutritional needs, social division of labor, and evolutionary adaptation. Although extensive studies have confirmed a general preference for sugars among ants, significant interspecific and intraspecific variation exists in the attractiveness of individual sugars [14]. Most ant species exhibit a distinct preference for sucrose, glucose, and fructose [32], reflecting an efficient energy-acquisition strategy likely evolved through long-term mutualism with honeydew-producing hemipterans. *Solenopsis invicta* is known to strongly prefer common sugars such as sucrose, melezitose, glucose, and fructose [15,33,34], though the exact preference hierarchy varies across reports. In this study, using a self-developed feeding preference assay system, we systematically evaluated the feeding preferences of *S. invicta* workers for ten sugar solutions and validated the findings with field attraction experiments. Both laboratory and field experimental results demonstrated that workers exhibited the strongest feeding and attraction preference for sucrose, with no statistically significant difference from glucose in either consumption or relative feeding intensity (Figure 3a–c). This finding aligns with the conclusions of Jaleel et al. [25] and Wen et al. [35]. It is noteworthy, however, that Zhou et al. [15] reported a significantly higher preference for melezitose over sucrose in *S. invicta* using indoor and outdoor two-choice tests. This discrepancy likely stems from methodological differences.

Sugar concentration is another key factor influencing ant feeding decisions. The attractiveness of sucrose to ants generally increases with its concentration [32]. Similarly, in *S. invicta*, worker recruitment declines with decreasing sugar concentration, regardless of whether colonies are monogyne or polygyne [36]. This study also confirms the concentration-dependent preference for sucrose in *S. invicta*. Our laboratory research showed that the relative feeding intensity trended upward with higher sucrose concentrations (Figure 4a). However, consumption after 60 min increased at a diminishing rate at higher concentrations, with consumption of the 70% solution even lower than that of 40% (Figure 4b). A similar phenomenon has been observed in other ant species. For instance, the intake of sugar by *Rhytidoponera metallica* workers was positively correlated with sugar concentration on the first day, while negatively correlated on the second day [37]. This is likely due to the increased viscosity of high-concentration sugar solutions, which can reduce liquid intake rates in ants [38,39]. In *S. invicta*, both crop load and intake rate decrease as sucrose concentration rises [40]. These results suggest a potential trade-off in the foraging strategy of worker ants. Although high-concentration sugar solutions offer greater energetic reward, their elevated viscosity may physically limit transport efficiency.

In addition to carbohydrates, ants also require protein-rich foods to meet their physiological needs. The regulation of carbohydrate to protein intake in ants is influenced by environmental conditions, colony reproductive status, food availability, and species-specific traits [22,23,41,42,43]. Since proteins are biological macromolecules composed of amino acids, a preference for proteinaceous food suggests a corresponding preference for specific amino acids. Therefore, investigating the feeding preference for amino acids by *S. invicta* can not only clarify its targeted foraging mechanism, but also aid in screening highly attractive compounds for bait development. Previous studies have reported varied responses to amino acids among ant species. For example, the tropical fire ant *Solenopsis geminata* prefers honeydew rich in amino acids [44], and even solutions containing only amino acids can attract ants [45]. The ant *Pseudomyrmex ferrugineus* shows a significant preference for solutions containing leucine, phenylalanine, proline, and valine, with a clear preference at higher concentrations [46]. Conversely, other studies report that amino acid solutions are not inherently attractive to ants [33], and that their appeal often depends on the presence of sugar [47]. Our study demonstrates for the first time that *S. invicta* exhibits a strong and specific preference for leucine in both laboratory feeding experiments and field attraction experiments, significantly higher than for the other ten amino acids (Figure 3d–f). L-leucine, an essential branched-chain amino acid, plays a crucial role in insect physiology. It regulates protein synthesis and growth through the mechanistic target of rapamycin complex 1 (mTORC1) signaling pathway, influences energy metabolism, and functions as a neuroactive substance [48]. Recent research has advanced the understanding of the role and mechanism of leucine in regulating animal growth and development [49,50]. For example, leucine promotes protein synthesis in juvenile white shrimp *Litopenaeus vannamei* through the TOR signaling pathway [51]. Social insects, such as the red imported fire ant, preferentially ingest leucine to activate this pathway, which may help meet the high demand for protein synthesis during larval development and queen egg-laying, thereby providing important nutritional support for colony growth. However, leucine alone is less attractive to *S. invicta* than sucrose (Figure 6a–c), positioning it as a potentially effective cofactor for optimizing bait formulations rather than a standalone attractant.

Building on the preference screening for single compounds, we further evaluated mixtures of Top 3 or Top 5 sugars, and a mixture of all eleven amino acids. Interestingly, *S. invicta* workers exhibited a significantly stronger field attraction to a single sucrose or leucine solution than to a multi-sugar mixture or a mixture of eleven amino acids (Figure 5c; Figure 5f), underscoring a specific foraging focus on these two nutrients. A substantial body of research indicates that various ant species prefer sugar-amino acid mixtures over single sugar solutions [32,44,47,52,53]. For example, the Japanese carpenter ant *Camponotus japonicus* exhibits a stronger feeding preference for a mixture of glycine and glucose than for glucose alone, and its gustatory receptor cells show a glycine concentration-dependent increase in response to glucose [54]. In our study, a sucrose-leucine mixture significantly enhanced the field attraction of *S. invicta* workers, outperforming single solutions of either sucrose or leucine (Figure 6c). However, the sucrose-leucine mixture did not produce a statistically significant enhancement in feeding preference under laboratory conditions (Figure 6a,b). This discrepancy likely stems from the 48 h starvation period imposed on test ants in the laboratory, which may affect the worker foraging motivation compared to field populations. Our field study results align with general findings that amino acids can enhance the attractiveness of sugars [32], whereas the laboratory findings are consistent with the reports where sugar-amino acid mixtures did not significantly outperform single-sugar treatment [45].

Methodological design is a crucial consideration in studies of ant feeding preference. Traditional approaches, such as isolating individual ants from their soil nests via the “water drop method” [55], can obtain clean and uniform ant colonies, but they may disrupt the natural behavioral state of ants, thereby compromising the ecological validity of feeding decisions [27]. To minimize behavioral disturbance and better approximate natural foraging conditions, the feeding preference assay system for terrestrial ants was developed. This system preserves the native soil nest structure, employs the entire colony as the experimental unit, and allows workers to forage autonomously via an insertion rod onto a foraging platform, thereby simulating the natural foraging process. The strong concordance between laboratory and field results obtained with this system confirms its reliability and ecological relevance for assessing the feeding preferences in *S. invicta*. Notably, when using a two-choice test approach, *S. invicta* exhibits a greater preference for melezitose over sucrose [15]. These methodological differences may explain the discrepancies with prior reports.

## 5. Conclusions

This study employed a self-developed feeding preference assay system to systematically evaluate the feeding and attraction preferences of *S. invicta* workers towards various sugars, amino acids, and their concentrations and combinations through both laboratory and field experiments. Results demonstrated that *S. invicta* workers exhibited a pronounced feeding preference for sucrose and leucine, with both preferences being concentration-dependent. Furthermore, a mixture of sucrose and leucine solutions significantly enhanced the field attraction of workers. Notably, our self-developed feeding preference assay system provides a new tool for assessing the feeding preferences of terrestrial ants under near-natural conditions. These findings systematically clarify the foraging strategy of *S. invicta* toward key honeydew nutrients. For practical application, we recommend a bait formulation combining 20% sucrose with 0.5–1% leucine as a core attractant, which is expected to substantially improve field trapping efficacy for *S. invicta* workers.

## Data Availability

The original contributions presented in this study are included in the article. Further inquiries can be directed to the corresponding authors.
